# rePROBE: Workflow for Revised Probe Assignment and Updated Probe-set Annotation in Microarrays

**DOI:** 10.1016/j.gpb.2020.06.007

**Published:** 2021-02-11

**Authors:** Frieder Hadlich, Henry Reyer, Michael Oster, Nares Trakooljul, Eduard Muráni, Siriluck Ponsuksili, Klaus Wimmers

**Affiliations:** 1 Research Institute for Farm Animal Biology (FBN), Institute of Genome Biology, D-18196 Dummerstorf, Germany; 2 Faculty of Agricultural and Environmental Sciences, University of Rostock, D-18059 Rostock, Germany

**Keywords:** Mapping, Microarray, Probe assignment, Probe-set annotation, Genomic database

## Abstract

Commercial and customized **microarrays** are valuable tools for the analysis of holistic expression patterns, but require the integration of the latest genomic information. This study provides a comprehensive workflow implemented in an R package (rePROBE) to assign the entire probes and to annotate the probe sets based on up-to-date genomic and transcriptomic information. The rePROBE package can be applied to available gene expression microarray platforms and addresses both public and custom databases. The revised **probe assignment** and updated **probe-set annotation** are applied to commercial microarrays available for different livestock species, *i.e.*, chicken (*Gallus gallus*; ChiGene-1_0-st: 443,579 probes and 18,530 probe sets), pig (*Sus scrofa*; PorGene-1_1-st: 592,005 probes and 25,779 probe sets), and cattle (*Bos Taurus*; BovGene-1_0-st: 530,717 probes and 24,759 probe sets), as well as available for human (*Homo sapiens*; HuGene-1_0-st) and mouse (*Mus musculus*; HT_MG-430_PM). Using current species-specific transcriptomic information (RefSeq, Ensembl, and partially non-redundant nucleotide sequences) and genomic information, the applied workflow reveals 297,574 probes (15,689 probe sets) for chicken, 384,715 probes (21,673 probe sets) for pig, 363,077 probes (21,238 probe sets) for cattle, 481,168 probes (23,495 probe sets) for human, and 324,942 probes (32,494 probe sets) for mouse. These are representative of 12,641, 15,758, 18,046, 20,167, and 16,335 unique genes that are both annotated and positioned for chicken, pig, cattle, human, and mouse, respectively. Additionally, the workflow collects information on the number of single nucleotide polymorphisms (SNPs) within respective targeted genomic regions and thus provides a detailed basis for comprehensive analyses such as expression quantitative trait locus (eQTL) studies to identify quantitative and functional traits. The rePROBE R package is freely available at https://github.com/friederhadlich/rePROBE.

## Introduction

Current breeding goals and selection criteria for livestock species go beyond the performance and carcass parameters and also consider the variation of functional traits [1], [2]. To identify genetically robust farm animals, respective experimental approaches often require information from the various ‘omics’ levels [3]. In fact, the use of expression data enables the generation or the verification of hypotheses on biological processes. Microarray experiments are a valuable tool for obtaining precise information about co-expressed genes and gene networks, interactions between genes and traits, and phenotypic variations. In fact, the large amount of publicly available biological data from microarray analyses stored in the Gene Expression Omnibus (GEO) [4] can also be used to generate new hypotheses.

Microarrays are essentially a collection of single-stranded DNA oligos called “probes”. For Affymetrix arrays, each probe counts 25 nt, which should be complementary to predefined genomic target regions. Approximately 15–27 probes are aggregated to a “probe set” to represent a certain transcript (main probes). Moreover, up to 30,000 control probes are used to verify amplification and hybridization (spiked-in bacteria probes), making the microarray a reliable tool for holistic transcriptomic analysis. However, the need to clarify the identity of microarray probes and probe sets has been demanded elsewhere [5], [6]. Holistic expression analyses require highly accurate annotation data to ensure the quality and reliability of a dataset. In fact, microarray annotations used to be updated routinely [7], [8], [9], since there is a steadily growing body of genomic knowledge including coding and non-coding sequences, genomic variations, gene functions, gene localizations, and regulatory mechanisms.

Nowadays, the RNA-sequencing (RNA-seq) approach employing next-generation sequencing (NGS) technologies has become popular for genome-wide transcriptome profiling and is an open system that offers opportunities to discover novel splice variants and certain fractions of the RNA [10], [11]. The microarray platform is a closed system based on existing knowledge about the genome sequence. As such, microarrays represent a high-throughput and labor-saving approach in combination with a robust out-of-the-box data analysis. For processing and downstream analyses, powerful tools have been developed to facilitate identification of differentially expressed genes and insights into functional enrichment [12], [13]. The combination of RNA-seq and microarray approaches is thus valued to obtain a comprehensive picture at the expression level [14].

Keeping pace with the current development in genomic research, we aimed to provide a workflow enabling 1) a reassignment of microarray probes including the correction for existing single nucleotide polymorphisms (SNPs) and 2) a user-friendly update based on the current reference annotation data. Specifically, any combination of public and custom databases with different ranking priorities can be included. The developed workflow is implemented in a publicly available R [15] package termed rePROBE and is described in detail for transcriptome analysis on the example of commercial microarrays of chicken, pig, cattle, human, and mouse, but is not limited to these.

## Method

In the first step of the workflow, the analysis focuses on the exclusion of a number of probes from the initial probe to probe-set assignment due to 1) multiple matches to chromosomal regions or 2) mismatches to genomic sequences. In the second step, the gene symbol annotation of probe sets is updated. To demonstrate the feasibility of the workflow, commercially available microarrays are reassigned and reannotated. Thus, Affymetrix microarrays (Santa Clara, CA) of ChiGene-1_0-st (*Gallus gallus*; 443,579 probes; 18,530 probe sets), PorGene-1_1-st (*Sus scrofa*; 592,005 probes; 25,779 probe sets), BovGene-1_0-st (*Bos Taurus*; 530,717 probes; 24,759 probe sets), HuGene-1_0-st (*Homo sapiens*; 824,740 probes; 28,869 probe sets), and HT_MG-430_PM (*Mus musculus*; 496,468 probes; 45,101 probe sets) are used. Here, respective amplification and hybridization controls are omitted. The exemplary procedure employs four different databases: two have a global nature (RefSeq and Ensembl), and the other two represent additional data sources [Nucleotide collection (NT) and RefSeq DNA database (DNA)]. Regarding the assignment of probes to probe sets, the workflow allows a ranking of the databases to define the priority level for each database. Accordingly, the same ranks are processed simultaneously, whereby a successful mapping excludes probes for subsequent rank processing. For chicken, pig, and cattle examples, RefSeq and Ensembl are given rank 1, while NT and DNA are assigned rank 2. For human and mouse arrays solely, DNA is assigned rank 2.

The workflow offers the opportunity to implement one or more public and custom databases in a user-friendly manner. For the species of interest, specific files for annotation, SNP variation, and genome reference are automatically retrieved using the rePROBE *prepare_data* function. Corresponding databases include RefSeq (Gallus_gallus-5.0 Release 103, Sscrofa11.1 Release 106, Bos_taurus_UMD_3.1.1 Release 105, GRCh38.p12 Release 108, and GRCm38.p4 Release 107; https://ftp.ncbi.nih.gov/genomes) and Ensembl (Gallus_gallus.GRCg6a, Sus_scrofa.Sscrofa11.1, Bos_taurus.ARS-UCD1.2, Homo_sapiens.GRCh38.p12, and Mus_musculus.GRCm38.p6; all Release 97; ftp://ftp.ensembl.org/pub). Alternatively, FASTA sequences from NT (accessed for chicken, pig, and cattle on 12 July 2019, http://www.ncbi.nlm.nih.gov/ncbisearch/; sequence type: nucleotide) and from RefSeq DNA database including corresponding SNP information from dbSNP are obtained and processed. This feature has also been implemented in the *prepare_data* function. Data retrieved from NT are restricted to the respective organism and include sequences ranging between 50 nt and 10,000 nt.

**Figure 1:**
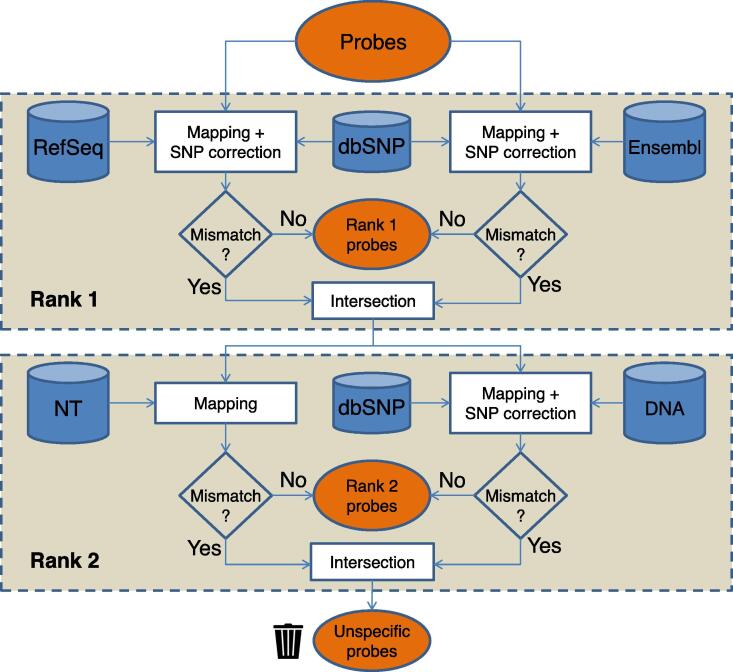
**Workflow to analyze probe sequences combining information from four different databases** All probes are initially mapped against RNA sequences retrieved from RefSeq and Ensembl (rank 1). Mapping results are corrected for known SNPs using information retrieved from dbSNP. Probes without mismatch in any tested RNA database are considered transcript-specific probes. Remaining probes which are perfectly mapped to the DNA sequences or any NT-derived sequences are considered genome-specific probes (rank 2). Probes which fail to be mapped to any of the source sequences are discarded and considered as unspecific probes. SNP, single nucleotide polymorphism; dbSNP, SNP database; NT, nucleotide collection; DNA, RefSeq DNA database.

The applied workflow for the probe classification using four different databases and two ranks is schematically shown in Figure 1. All mapping analyses are performed with the R package Rbowtie (v1.24.0) using the Bowtie short read aligner as originally presented by Langmead and colleagues [16]. Parameters are set to ‘-y --best --strata –a’ and ‘v = 2’, allowing up to two mismatches. Firstly, initial probe sequences are mapped to sequences retrieved from rank 1 database(s). If multiple databases are used, the individual genomic reference can be defined (*e.g.*, RefSeq and Ensembl). Mapping results that exhibit an identical genomic position (*e.g.*, transcript variants) are aggregated. Furthermore, probes assigned to different genomic positions are discarded. After correcting for SNPs, perfect matching probes are considered unique and specific in terms of rank 1.

**Figure 2:**
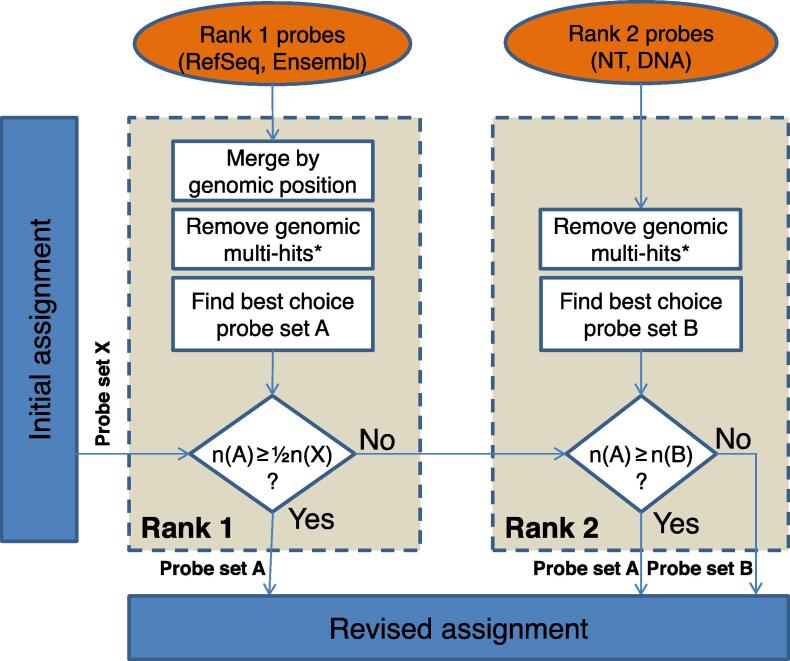
**Workflow applied to each probe set** Each of the probe sets obtained from the initial assignment is processed individually (probe set X). The first step comprises only transcript-specific probes of each probe set, which are uniquely mapped to the exonic part of the genome. The transcript variants are merged by genomic position. Sharing a single gene annotation prompts an assignment as probe set A (prominent role of gene symbols). Transcript-specific probes are used in the revised assignment if at least 50 % of the probes initially assigned to a certain probe set are present. Otherwise, probes with only unique genomic mappings to any alternative database are assigned as probe set B containing either an NT annotation or, if more dominating, a unique DNA region. Subsequently, the dominating probe set (containing more probes, *i.e.*, either probe set A or probe set B) is used for the revised assignment. *, “remove genomic multi-hits” is only applied for known chromosomes (1, 2, …, MT, X, Y). n, the number of probes in a probe set.

The information obtained from the *prepare_data* function is used to revise the assignment of probes to probe sets and, ultimately, to update the annotation of probe sets by using the rePROBE *run* function. Specifically, generated mapping information retrieved for rank 1 is evaluated as depicted in Figure 2. If the probes of rank 1 represent ≥50% of the probes initially assigned in the respective probe set, the remaining probes are discarded and will not be processed in terms of rank 2 databases. Probes which have not been successfully assigned according to rank 1 processing are mapped to NT sequences allowing no mismatches (‘v = 0’ due to missing SNP information) and to DNA sequences allowing SNP correction of up to two mismatches (Figure 1, lower panel). Reverse orientation of sequences is allowed only for DNA entries. Perfect alignments are considered as rank 2 probes. Probes, which fail to be aligned to any of the source sequences or which are defined as uninformative based on the probe-to-probe-set assignment, are considered as unspecific probes. Hence, an initially defined probe set might 1) contain entirely rank 1 specific probes, 2) contain rank 2 information to approximate the best choice assignment, or 3) be completely removed. For the annotation of probes and probe sets, current information is retrieved from the corresponding databases and compiled in comprehensive probe and probe-set annotation files, suitable for numerous subsequent analyses.

## Application and reporting formats

**Figure 3:**
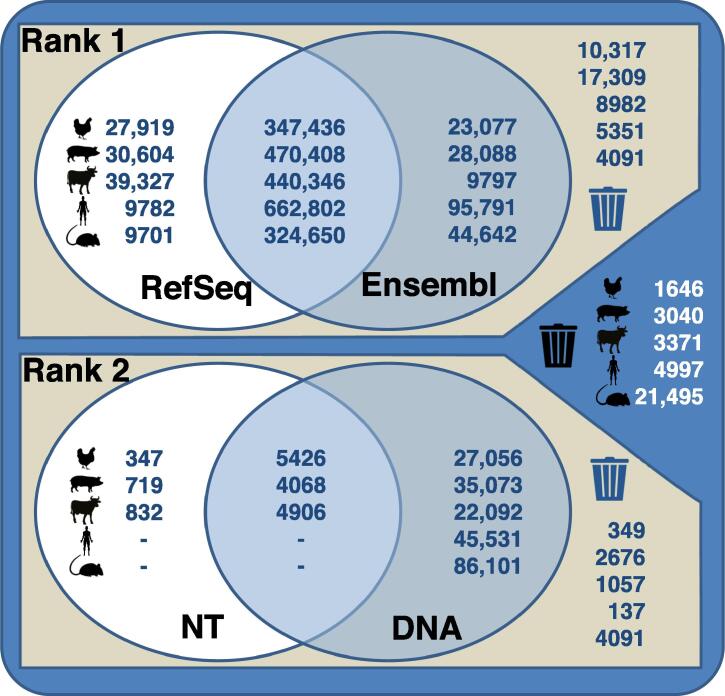
**Probe mapping information using specific genomic databases for chicken, pig, cattle, human, and mouse microarrays** Generated mapping information retrieved for rank 1 (RefSeq, Ensembl) and rank 2 (NT, DNA) databases is displayed as Venn diagrams. For the exemplary microarrays, the mapping reveals a number of probes which are identified by one or two databases, as well as probes which fail to be aligned to any of the source sequences.

The assignment of successfully mapped probes is shown in Figure 3. Specifically, chicken mapping information retrieved from various databases revealed 89.8% of the spotted probes to be rank 1 probes (pig: 89.4%; cattle: 92.2%; human: 93.2%; mouse: 76.3%); 87.2% of these probes matched transcript sequences retrieved from both RefSeq and Ensembl (pig: 88.9%; cattle: 90.0%; human: 86.3%; mouse: 85.7%). Ultimately, the revised probe assignment which keeps only probes of best choice probe sets relies on 67.1% of all given probes for the chicken chip (pig: 65.0%; cattle: 68.4%; human: 58.3%; mouse: 65.5%). The revised assignment, therefore, used a subset of the spotted information on the various microarrays. The reliability of the information obtained at the probe level has, therefore, been considerably improved. This probe information includes details of the genomic mapping position, exon position, and the number of putative SNP positions. The integration of these data at the probe level enables the usage of the microarrays for in-depth analyses such as the investigation of transcript and splice variants.

The revised probe-set assignment substantially benefits from the available genomic information to ensure the measurement of specific targets. In the chicken microarray, 12,581 probe sets remained, containing ≥50% of their initially affiliated probes (pig: 17,374; cattle: 17,199; human: 18,036; mouse: 30,050). A total of 8815 probe sets were entirely unaffected and corresponded to the initial assignment (pig: 10,661; cattle: 13,074; human: 13,335; mouse: 23,522). However, the revised probe-to-probe-set assignment identified 2841 probe sets, which comprised exclusively unspecific probes (pig: 4106; cattle: 3521; human: 5601; mouse: 12,607).

**Figure 4:**
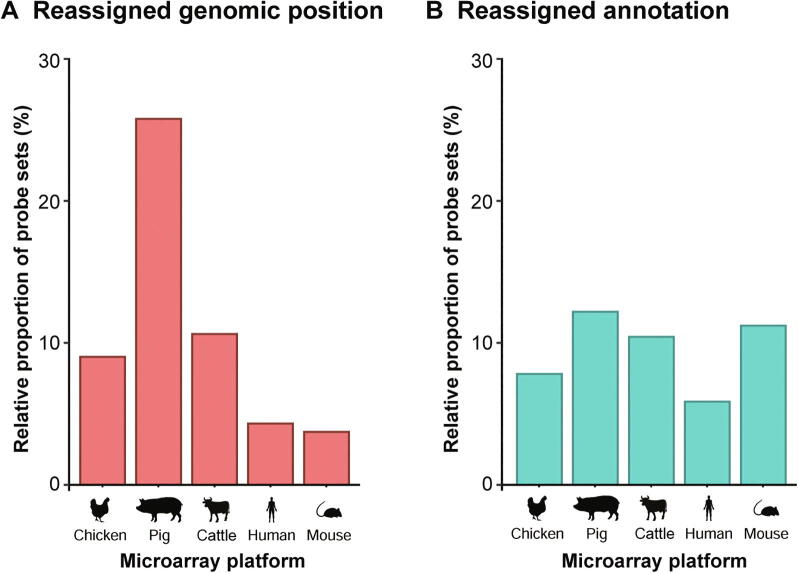
**Reassignment of genomic positions and new annotations at the probe-set level exemplified for chicken, pig, cattle, human, and mouse** The performance of rePROBE in reassigning microarray probe sets to genomic positions (**A**) and reannotating probe sets (**B**) based on increasingly accessible genomic knowledge was evaluated for commercially available microarrays of chicken (ChiGene-1_0-st), pig (PorGene-1_1-st), cattle (BovGene-1_0-st), human (HuGene-1_0-st), and mouse (HT_MG-430_PM). Displayed are the relative proportions of reassigned and reannotated probe sets.

According to the current genomic knowledge, the applied workflow for the chicken microarray revealed 15,689 probe sets representing 12,641 unique genes to be both annotated and positioned (pig: 21,673 probe sets / 15,758 unique genes; cattle: 21,238 probe sets / 18,046 unique genes; human: 23,495 probe sets/ 20,167 unique genes; mouse: 32,494 probe sets / 16,335 unique genes) (Tables S1–S3). The respective gene symbols were retrieved from 1) both RefSeq and Ensembl (chicken: 8159; pig: 11,675; cattle: 12,563; human: 11,540; mouse: 18,497), 2) RefSeq (chicken: 3306; pig: 3948; cattle: 5157; human: 3319; mouse: 912), 3) Ensembl (chicken: 1870; pig: 2662; cattle: 1129; human: 6123; mouse: 4536), 4) both NT and DNA (chicken: 478; pig: 426; cattle: 395), and 5) NT (chicken: 36; pig: 84; cattle: 58) entries. The number of probe sets, from which both genomic positions (Figure 4A) and annotations (Figure 4B) are currently known, reflects the improvement in accessible genomic knowledge since the initial microarray design.

The applied workflow combined genomic information and gene annotations, which have been retrieved from various databases. Ranks can be used to define a specific priority for each database. Accordingly, the user can define the most actual and reliable database(s) as primary source for assignment and annotation. Nevertheless, the workflow allows to further implement other databases as well as own sequence information if available in a user-defined ranking scheme. This approach has proven to be beneficial in terms of the detectability of genes and transcript variants and the overall reliability of expression analyses [17].

## Implementation

The revised microarray workflow is implemented in the R package rePROBE (https://github.com/friederhadlich/rePROBE). rePROBE runs in the R environment under both Windows and Linux operation systems. It provides an easy-to-use text-based user interface. The *prepare_data* function comprises different R functions to automatically provide and prepare the needed information based on the initial array definition files. The interactive user interface queries basic requirements, including working directory, project name, species, and the target databases. Subsequently, the revised probe-set assignment is prepared via the *run* function, which provides an R environment and data tables for downstream microarray analysis. Revised probe assignment and probe-set annotation for the ChiGene-1_0-st (*Gallus gallus*), PorGene-1_1-st (*Sus scrofa*), and BovGene-1_0-st (*Bos taurus*) microarrays are provided (Tables S1–S3). The required computing time per species (15 CPUs, desktop-PC) is ∼2–3 h and includes indexing, probe assignment, and probe-set annotation. A summary concerning the applied microarray platform can be accessed via the *show_report* function.

## Applications in transcriptional research

The application of the workflow generates information on genomic and exonic positions, polymorphisms, and probe assignment. In order to enable an interrelation with the vast majority of databases and genomic resources, the annotation refers to various transcript identifiers, including HUGO gene symbols and Ensembl IDs.

Regarding the outlined examples, the revised probe assignment and updated probe-set annotation have proven to be applicable for the ChiGene-1_0-st, PorGene-1_1-st, and BovGene-1_0-st microarrays. In addition, the workflow for the HuGene-1_0-st and HT_MG-430_PM microarrays was successfully applied (Table S4). The workflow will contribute to describing a sophisticated picture of quantitative and functional traits in the species of interest. The physical redesign of microarray platforms will likewise improve the association between expression levels and corresponding transcripts [18]. Using microarray-derived transcriptional data as phenotypes will be beneficial in identifying expression quantitative trait loci (eQTLs) towards a sophisticated selection of candidate genes related to a trait in question [19], [20].

The revised assignment according to up-to-date information will improve data quality as previously shown for different species [21]. However, the annotation level clearly depends on the probe-set design [22]. Probes are preferentially designed against the 3′ untranslated regions (UTRs) of transcripts. Respective sequence information is mostly derived by experimental evidence, which does not necessarily represent the complete transcript sequence and may not be fully implemented in the current databases [22]. Therefore, the exclusion of unspecific probes according to the current database knowledge is indispensable as it substantially reduces the error due to cross-hybridizations at the probe level [23]. However, the partial mapping of probes especially represented by the number of genome-specific probes requires a sequential update of the assignment and annotation with a special emphasis on the growing knowledge of SNPs in the era of NGS. Hence, detailed information related to the probe-specific occurrence of SNPs is provided (Tables S1–S3).

## Code availability

rePROBE is freely available at GitHub (https://github.com/friederhadlich/rePROBE). All information regarding installation and application of the tool is provided.

## Competing interests

The authors have declared no competing interests.

### CRediT authorship contribution statement

**Frieder Hadlich:** Conceptualization, Software, Formal analysis, Visualization. **Henry Reyer:** Conceptualization, Formal analysis, Writing – original draft. **Michael Oster:** Conceptualization, Formal analysis, Writing – original draft. **Nares Trakooljul:** Supervision. **Eduard Muráni:** Writing – review & editing, Supervision. **Siriluck Ponsuksili:** Resources, Writing – review & editing, Supervision. **Klaus Wimmers:** Writing – review & editing, Supervision, Funding acquisition.

## Supplementary material

Supplementary Table S1 **Information related to the probe sets represented in the chicken microarray (ChiGene-1_0-st) after using rePROBE**

Supplementary Table S2 **Information related to the probe sets represented in the pig microarray (PorGene-1_1-st) after using rePROBE**

Supplementary Table S3 **Information related to the probe sets represented in the cattle microarray (BovGene-1_0-st) after using rePROBE**

Supplementary Table S4 **Summary of performance indicators referring to the applied exemplary microarray platforms reassigned and reannotated with rePROBE**
