# Supplementary material for: rePROBE: Workflow for Revised Probe Assignment and Updated Probe-set Annotation in Microarrays
Source: Genomics Proteomics Bioinformatics. 2021 Feb 11;19(6):1043–9. doi: 10.1016/j.gpb.2020.06.007 (PMC9402582; doi:10.1016/j.gpb.2020.06.007)
Supplement: Supplementary Table S4 — Summary of performance indicators referring to the applied exemplary microarray platforms reassigned and reannotated with rePROBE [file mmc4.docx]

**Table S4 Summary of performance indicators referring to the applied exemplary microarray platforms reassigned and reannotated with rePROBE**

| **Chip** | **Number of probes** | **Number of probe sets** | **Number of mapped probes rank 1** | **Number of mapped probes rank 2** | **Number of qualified probes** | **Number of qualified probe sets** |
| --- | --- | --- | --- | --- | --- | --- |
| Gene-1_0-st-v1 | 824,740 | 28,869 | 768,721 | 45,528 | 478,022 | 23,495 |
| Mouse430_2 | 496,468 | 45,101 | 378,993 | 85,972 | 323,085 | 32,494 |
| ChiGene-1_0-st | 443,579 | 18,530 | 398,432 | 28,754 | 295,631 | 15,689 |
| PorGene-1_1-st | 592,005 | 25,779 | 529,100 | 34,899 | 380,142 | 21,673 |
| BovGene-1_0-st | 530,717 | 24,759 | 489,470 | 23,844 | 359,370 | 21,238 |

*Note*: Commercial microarrays were used for chicken (ChiGene-1_0-st), pig (PorGene-1_1-st), cattle (BovGene-1_0-st), human (HuGene-1_0-st), and mouse (HT_MG-430_PM).
